# Targeted next-generation sequencing for detection of *PIK3CA* mutations in archival tissues from patients with Klippel–Trenaunay syndrome in an Asian population

**DOI:** 10.1186/s13023-023-02893-1

**Published:** 2023-09-04

**Authors:** Yuki Sasaki, Kosuke Ishikawa, Kanako C. Hatanaka, Yumiko Oyamada, Yusuke Sakuhara, Tadashi Shimizu, Tatsuro Saito, Naoki Murao, Tomohiro Onodera, Takahiro Miura, Taku Maeda, Emi Funayama, Yutaka Hatanaka, Yuhei Yamamoto, Satoru Sasaki

**Affiliations:** 1 Department of Plastic and Reconstructive Surgery, Faculty of Medicine, Graduate School of Medicine, Hokkaido University, Kita 15, Nishi 7, Kita‐ku, Sapporo, 060-8638 Japan; 2 Center for Vascular Anomalies, Department of Plastic and Reconstructive Surgery, Tonan Hospital, Hokkaido, Japan; 3 Center for Development of Advanced Diagnostics, Institute of Health Science Innovation for Medical Care, Hokkaido University Hospital, Hokkaido, Japan; 4 Department of Diagnostic Pathology, Tonan Hospital, Hokkaido, Japan; 5 Department of Diagnostic and Interventional Radiology, Tonan Hospital, Hokkaido, Japan; 6 Research Division of Genome Companion Diagnostics, Hokkaido University Hospital, Hokkaido, Japan; 7 Riken Genesis Co., Ltd, Tokyo, Japan; 8 Department of Orthopedic Surgery, Faculty of Medicine, Graduate School of Medicine, Hokkaido University, Hokkaido, Japan

**Keywords:** Capillary malformations, High-throughput nucleotide sequencing, Klippel–Trenaunay Syndrome, Limb hypertrophy, Lymphatic abnormalities, Phosphatidylinositol 3-Kinase, *PIK3CA*-related overgrowth spectrum, Segmental hypertrophy, Vascular malformations, Venous malformations

## Abstract

**Background:**

Klippel–Trenaunay syndrome (KTS) is a rare slow-flow combined vascular malformation with limb hypertrophy. KTS is thought to lie on the *PIK3CA*-related overgrowth spectrum, but reports are limited. *PIK3CA* encodes p110α, a catalytic subunit of phosphatidylinositol 3-kinase (PI3K) that plays an essential role in the PI3K/AKT/mammalian target of rapamycin (mTOR) signaling pathway. We aimed to demonstrate the clinical utility of targeted next-generation sequencing (NGS) in identifying *PIK3CA* mosaicism in archival formalin-fixed paraffin-embedded (FFPE) tissues from patients with KTS.

**Results:**

Participants were 9 female and 5 male patients with KTS diagnosed as capillaro-venous malformation (CVM) or capillaro-lymphatico-venous malformation (CLVM). Median age at resection was 14 years (range, 5–57 years). Median archival period before DNA extraction from FFPE tissues was 5.4 years (range, 3–7 years). NGS-based sequencing of *PIK3CA* achieved an amplicon mean coverage of 119,000x. *PIK3CA* missense mutations were found in 12 of 14 patients (85.7%; 6/8 CVM and 6/6 CLVM), with 8 patients showing the hotspot variants E542K, E545K, H1047R, and H1047L. The non-hotspot *PIK3CA* variants C420R, Q546K, and Q546R were identified in 4 patients. Overall, the mean variant allele frequency for identified *PIK3CA* variants was 6.9% (range, 1.6–17.4%). All patients with geographic capillary malformation, histopathological lymphatic malformation or macrodactyly of the foot had *PIK3CA* variants. No genotype–phenotype association between hotspot and non-hotspot *PIK3CA* variants was found. Histologically, the vessels and adipose tissues of the lesions showed phosphorylation of the proteins in the PI3K/AKT/mTOR signaling pathway, including p-AKT, p-mTOR, and p-4EBP1.

**Conclusions:**

The PI3K/AKT/mTOR pathway in mesenchymal tissues was activated in patients with KTS. Amplicon-based targeted NGS could identify low-level mosaicism from low-input DNA extracted from FFPE tissues, potentially providing a diagnostic option for personalized medicine with inhibitors of the PI3K/AKT/mTOR signaling pathway.

**Supplementary Information:**

The online version contains supplementary material available at 10.1186/s13023-023-02893-1.

## Background

Klippel–Trenaunay syndrome (KTS, [MIM 149,000]) is a slow-flow combined vascular malformation with a characteristic triad of symptoms: capillary malformation (CM), limb hypertrophy, and venous malformation (VM) with or without lymphatic malformation (LM) [1, 2]. From 2012 onward, several studies have reported on *PIK3CA* variants found in KTS. Kurek et al. screened DNA extracted from lesional tissue in 3 of 15 patients with KTS and found *PIK3CA* variants [3]. Luks et al. reported that up to 90% of patients with KTS have *PIK3CA* variants in pathological lesions. Accordingly, KTS is thought to lie on the *PIK3CA*-related overgrowth spectrum (PROS) [4, 5], but reports are limited [3, 6–9] and genetic differences among races are unknown.

*PIK3CA* encodes p110α, a catalytic subunit of phosphatidylinositol 3-kinase (PI3K) that plays a role in cellular processes such as proliferation, motility, invasion, and death through its involvement in the PI3K/AKT/mammalian target of rapamycin (mTOR) signaling pathway [10]. Moreover, p110α is required for endothelial cell migration during angiogenesis [11, 12], and its aberrant activation has been associated with the development of vascular malformations [13, 14]. Activation of PI3K leads to phosphorylation of AKT followed by mTOR and its downstream targets, including eukaryotic translation initiation factor 4E-binding protein 1 (4EBP1) [15].

The activation of mutations in *PIK3CA* is reported to play a role in many human cancers [16]. Of the *PIK3CA* variants, more than 80% are found at three hotspots: the glutamates E542 and E545, located in the helical domain of exon 10, and the histidine H1047, located in the kinase domain of exon 21 [17]. These three mutations exert the strongest effect on downstream signaling and enzymatic activation [17]. In patients with KTS as well, E542K, E545K, H1047R, and H1047L are the most frequent (i.e., hotspot) variants [3, 6, 8, 9].

These somatic gain-of-function variants, which arise in the postzygotic stage during embryonic development, result in a mosaic pattern in the affected lesion [5]. Therefore, molecular testing of peripheral blood or saliva has been ineffective for detecting pathogenic variants in patients with PROS [3]. Meanwhile, archival formalin-fixed paraffin-embedded (FFPE) tissues can be a valuable resource for clinical genomic studies [18], but the DNA obtained from these tissues can have a wide range of quality depending on factors such as age, DNA–protein crosslinking, fixation conditions, and inhibitors, all of which can affect downstream genomic analyses [19].

Next-generation sequencing (NGS) involving an amplicon-based targeted sequencing method with high sensitivity can identify low-level mosaicism from low-input DNA extracted from FFPE tissues and provide a diagnostic option when affected tissue is available [20]. Therefore, this study aimed to demonstrate the clinical utility of targeted NGS with a custom-designed panel for identifying *PIK3CA* mosaicism in archival FFPE tissues from patients with KTS, a relatively rare vascular malformation with limb hypertrophy.

## Results

### Patient characteristics

**Fig. 1:**
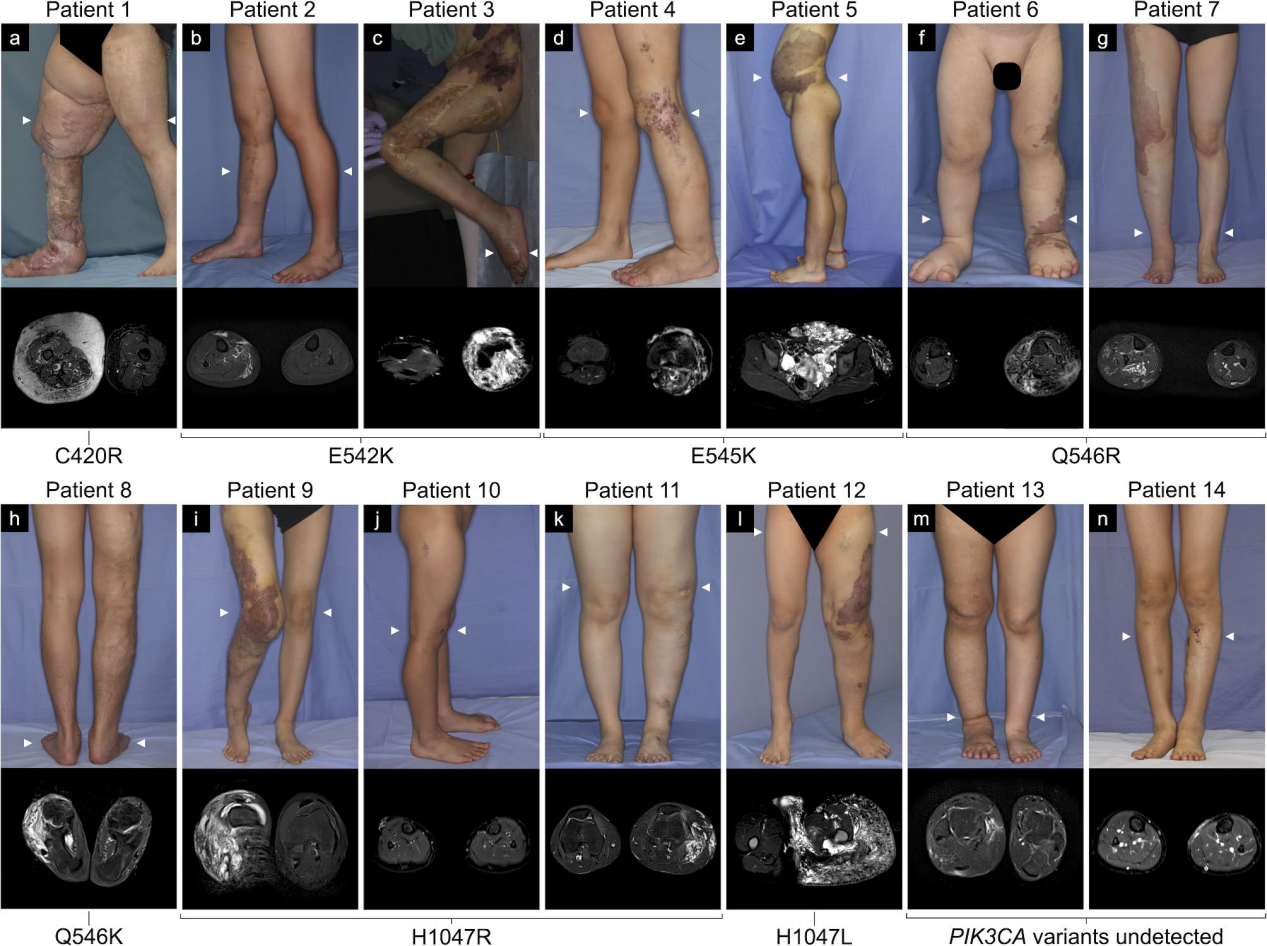
Clinical photographs (upper) and short-tau inversion recovery or fat-suppressed T2-weighted magnetic resonance images (lower) of the patients with Klippel–Trenaunay syndrome with detected or undetected *PIK3CA* variants. Various clinical manifestations were observed, including geographic capillary malformations (**a**, **c**-**g**, **i**, **l**), lower limb discrepancy in terms of length (**c**, **d**, **f**, **g**, **i**, **l**, **m**), and macrodactyly (**a**, **c**, **d**, **f**, **l**). Magnetic resonance images are axial views at the arrowhead position in each clinical photograph with the lesions of high signal intensity

Participants were 9 female and 5 male patients, including 5 (35.7%) adults (defined as age 18 years or older). Their clinical characteristics and genetic profiles are shown in Table 1. Median age at resection was 14 years (range, 5–57 years). Archival median period before DNA extraction from FFPE tissues was 5.4 years (range, 3–7 years). Lesions were resected from the abdomen (*n* = 2), buttock (*n* = 1), thigh (*n* = 2), knee (*n* = 3), lower leg (*n* = 4), and foot (*n* = 2). Tissue specimens were skin with subcutaneous tissue (*n* = 8), subcutaneous tissue only (*n* = 5), and subcutaneous tissue with muscles (*n* = 1). Figure 1 shows clinical photographs and magnetic resonance imaging (MRI) of all patients with detected *PIK3CA* variants. Patient 1 [21], patients 3 and 4 [22], and patient 9 [23] were previously reported without genetic analyses.

**Table 1:** Summary of clinical manifestations and identified *PIK3CA* variants in patients with Klippel–Trenaunay syndrome

Patient	Sex	Age (years)*	Lesions resected	Specimen	Identified variants	VAF	Diagnoses	Types of CM	Lower limb discrepancy in		Macrodactyly	Previous treatments
									Length	Girth		
1	M	36	Thigh	Skin, SC	C420R	1.6%	CLVM	Geographic	−	Rt > Lt	Rt	PR, Sc
2	F	14	Abdomen	Skin, SC	E542K	3.3%	CLVM	−	−	Rt > Lt	−	PR, Sc, TAE
3	F	57	Lower leg	Skin, SC	E542K	10.9%	CVM	Geographic	Lt > Rt	Lt > Rt	Lt	PR, Sc
4	F	6	Knee	Skin, SC	E545K	7.5%	CLVM	Geographic	Lt (ED) > Rt	Lt > Rt	Lt	PR, HL, Sc, TAE
5	F	16	Abdomen	SC	E545K	12.7%	CLVM	Geographic	−	Lt > Rt	−	PR, Sc, TAE
6	M	7	Lower leg	SC	Q546R	3.9%	CLVM	Geographic	Rt (ED) > Lt	Lt > Rt	Bilateral	PR, HL, Sc
7	F	26	Foot	SC	Q546R	5.9%	CVM	Geographic	Rt > Lt	Rt > Lt	−	HL, Sc
8	M	25	Foot	SC	Q546K	2.3%	CVM	−	−	Rt > Lt	−	Sc
9	F	17	Knee	Skin, SC	H1047R	17.4%	CVM	Geographic	Rt > Lt	Rt > Lt	−	PR, Sc, TAE
10	F	5	Knee	Skin, SC	H1047R	1.6%	CVM	−	−	Rt > Lt	−	−
11	F	22	Thigh	SC, M	H1047R	8.1%	CVM	−	−	Lt > Rt	−	PR, Sc
12	M	11	Buttock	Skin, SC	H1047L	7.8%	CLVM	Geographic	Lt > Rt	Lt > Rt	Lt	PR, Sc, TAE
13	F	13	Lower leg	SC	−	−	CVM	−	Rt > Lt	Rt > Lt	−	HL, Sc, TAE
14	M	11	Lower leg	Skin, SC	−	−	CVM	−	−	Lt > Rt	−	PR, Sc

*Age at resection; SC, subcutaneous tissue; M, muscle; VAF, variant allele frequency; CM, capillary malformation; CVM, capillaro-venous malformation; CLVM, capillaro-lymphatico-venous malformation; Lt, left; Rt, right; ED, epiphysiodesis; PR, partial resection; Sc, sclerotherapy; TAE, transcatheter arterial embolization; HL, high ligation of a lateral marginal vein in the thigh

Clinicopathological diagnoses were capillaro-venous malformation (CVM) (*n* = 8) and capillaro-lymphatico-venous malformation (CLVM) (*n* = 6). Geographic CMs were found in 8 patients. Lower limb discrepancy (LLD) in terms of length was found in 7 patients, including 2 patients with epiphysiodesis, while LLD in terms of girth was observed in all 14 patients (right-sided hypertrophy in 7 patients). Digital anomalies were found in 5 patients as macrodactyly of the foot. Patient 7 had bilateral lesions in the lower limbs (with CLVM in the left limb) and bilateral macrodactyly, and the longer right limb required epiphysiodesis. Thirteen patients had received treatment prior to resection for genetic analysis, including partial resection (*n* = 10), percutaneous sclerotherapy (*n* = 12), transcatheter arterial embolization for micro-arteriovenous shunts (*n* = 6), and high ligation of a lateral marginal vein in the thigh (*n* = 4).

### Detection of ***PIK3CA*** variants

Median DNA concentration from FFPE tissues measured using a Qubit 3.0 Fluorometer and a 2200 TapeStation system were respectively 20.5 ng/µL and 25.5 ng/µL. NGS-based ultradeep sequencing of *PIK3CA* achieved an amplicon mean coverage of 119,000x (range, 96,000–142,000x) for FFPE tissues and 107,000x (range, 95,000–136,000x) for controls.

**Fig. 2:**
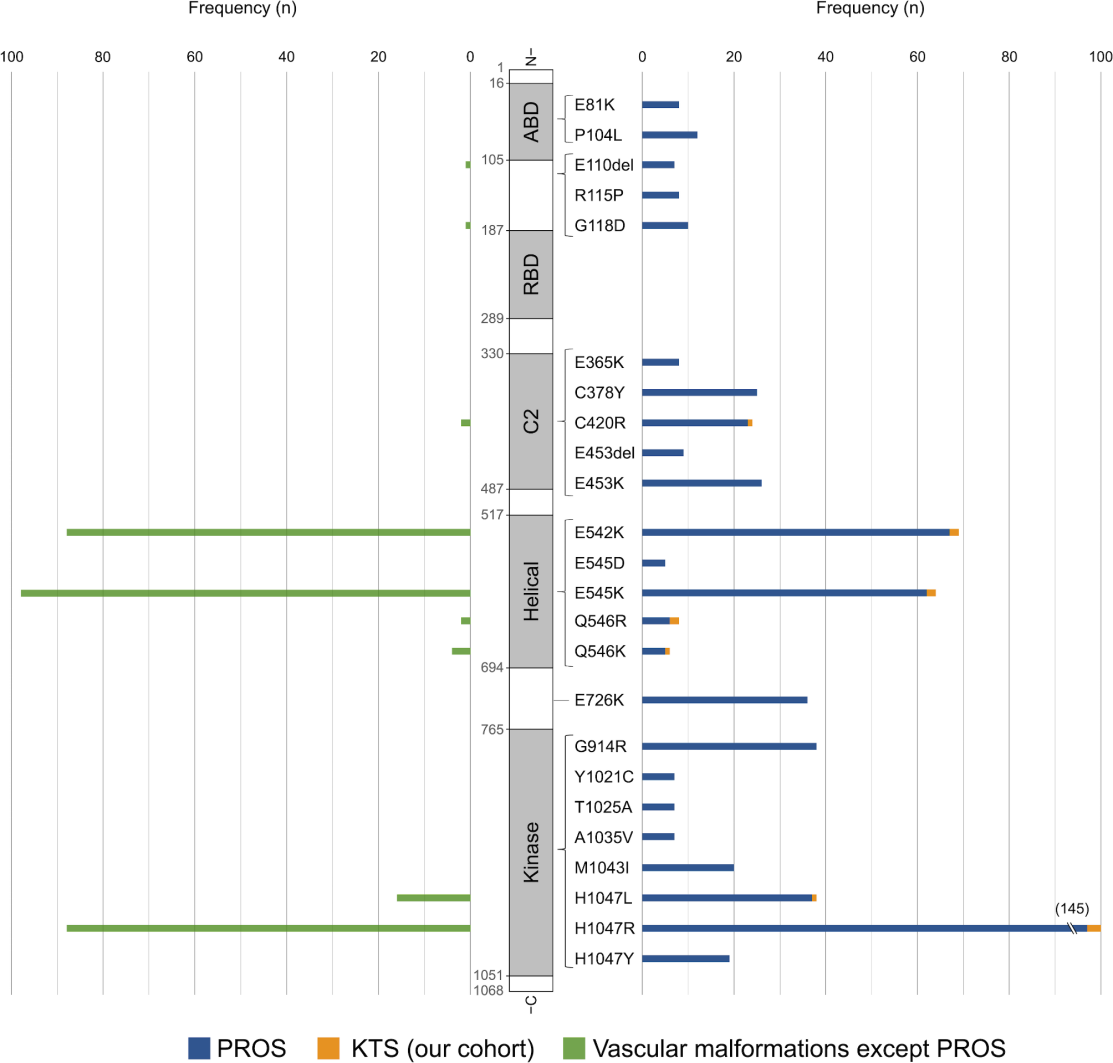
Distribution of frequent *PIK3CA* variants in *PIK3CA*-related overgrowth spectrum (PROS) [3, 6–8, 20, 24–48] and vascular malformations [6, 8, 42, 49–55] from the literature as well as our cohort (variants in PROS ≥ 5 patients in each variant). Right, variants found in patients with PROS (blue, *n* = 597) in the literature and Klippel–Trenaunay syndrome (KTS) in our cohort (orange, *n* = 12). Left, variants found in patients with vascular malformations except PROS (green, *n* = 300) in the literature. ABD, p85α-binding domain; RBD, Ras-binding domain; C2, C2 domain; Helical, helical domain; Kinase, kinase domain

The detected *PIK3CA* variant frequency in positive control DNA of 5%, 1%, 0.5%, and 0.1% of E545K/ H1047R mixture were respectively 4.3%, 0.8%, 0.5%, and 0.1% in E545K and 10.3%, 1.2%, 0.5%, and 0.1% in H1047R. No *PIK3CA* variants were detected in negative control DNA. *PIK3CA* missense mutations were found in 12 of 14 patients (85.7%; 6/8 CVM and 6/6 CLVM) (Table 1). Of the 12 variants detected, 8 (66.7%) were hotspot variants: E542K (c.1624G > A) in 2 patients, E545K (c.1633G > A) in 2, H1047R (c.3140 A > G) in 3, and H1047L (c.3140 A > T) in 1. The rest were 3 distinct non-hotspot variants: C420R (c.1258T > C) in 1 patient, Q546R (c.1637 A > G) in 2, and Q546K (c.1636 C > A) in 1. All of the detected *PIK3CA* variants were previously reported in patients with PROS or LM and are considered pathogenic variants according to ClinVar (Table 2). Overall, the mean variant allele frequency (VAF) for the identified *PIK3CA* variants was 6.9% (range, 1.6–17.4%). Summaries of the *PIK3CA* variants in PROS (*n* = 696) [3, 6–8, 20, 24–48] and vascular malformations except PORS (including LM, VM, fibro-adipose vascular anomaly and combined vascular malformations; *n* = 307) [6, 8, 42, 49–55] from the literature as well as our cohort (*n* = 12) are presented in Additional file 1, Fig. 2 (frequent variants in PROS ≥ 5 patients in each variant, *n* = 597 from the literature; vascular malformations except PORS, *n* = 300), and Table 2 (variants presented in Fig. 2), including the ranks in COSMIC v97 and variant class in ClinVar.

**Table 2:** Summary of the frequent *PIK3CA* variants found in PROS and vascular malformations

Amino acid variants	Ranks in COSMIC^1^	Counts^1^	Relative frequency^2^	Variant class^3^
H1047R^a^	1	5,368	36.73	Pathogenic
E545K^a^	2	4,111	28.13	Pathogenic
E542K^a^	3	2,515	17.21	Pathogenic
H1047L^a^	4	739	5.06	Pathogenic
Q546K^b^	8	301	2.06	Conflicting interpretations of pathogenicity
C420R^b^	9	258	1.77	Pathogenic
M1043I	11	190	1.30	Pathogenic/Likely pathogenic
E726K	12	168	1.15	Pathogenic
Q546R^b^	13	150	1.03	Pathogenic
H1047Y	14	143	0.98	Pathogenic
G118D	15	124	0.85	Pathogenic
E81K	17	113	0.77	Pathogenic
E453K	19	98	0.67	Pathogenic
Y1021C	25	77	0.53	Pathogenic
T1025A	31	60	0.41	Pathogenic/Likely pathogenic
E545D	34	58	0.40	Pathogenic/Likely pathogenic
E110del	42	46	0.31	Likely pathogenic
E365K	54	34	0.23	Pathogenic
P104L	63	26	0.18	Pathogenic/Likely pathogenic
A1035V	113	11	0.08	Pathogenic
G914R	127	9	0.06	Pathogenic
R115P	159	6	0.04	Likely pathogenic
C378Y	159	6	0.04	Pathogenic
E453del	177	5	0.03	Pathogenic
Total *PIK3CA* vaiants		14,616	100	

The *PIK3CA* variants found in *PIK3CA*-related overgrowth spectrum (PROS) [3, 6–8, 20, 24–48] and vascular malformations [6, 8, 42, 49–55]. ^a^Hotspot variants. ^b^Non-hotspot variants detected in our cohort. ^1^Ranks and counts in COSMIC (Catalogue of Somatic Mutations in Cancer) v97. ^2^Relative frequency in this table. ^3^Variant class in ClinVar.

### Genotype–phenotype analysis

All patients with geographic CM, histopathological LM, or macrodactyly of the foot had *PIK3CA* variants. However, we did not find any association with phenotype or its severity between hotspot and non-hotspot *PIK3CA* variants.

### Histopathological analysis

**Fig. 3:**
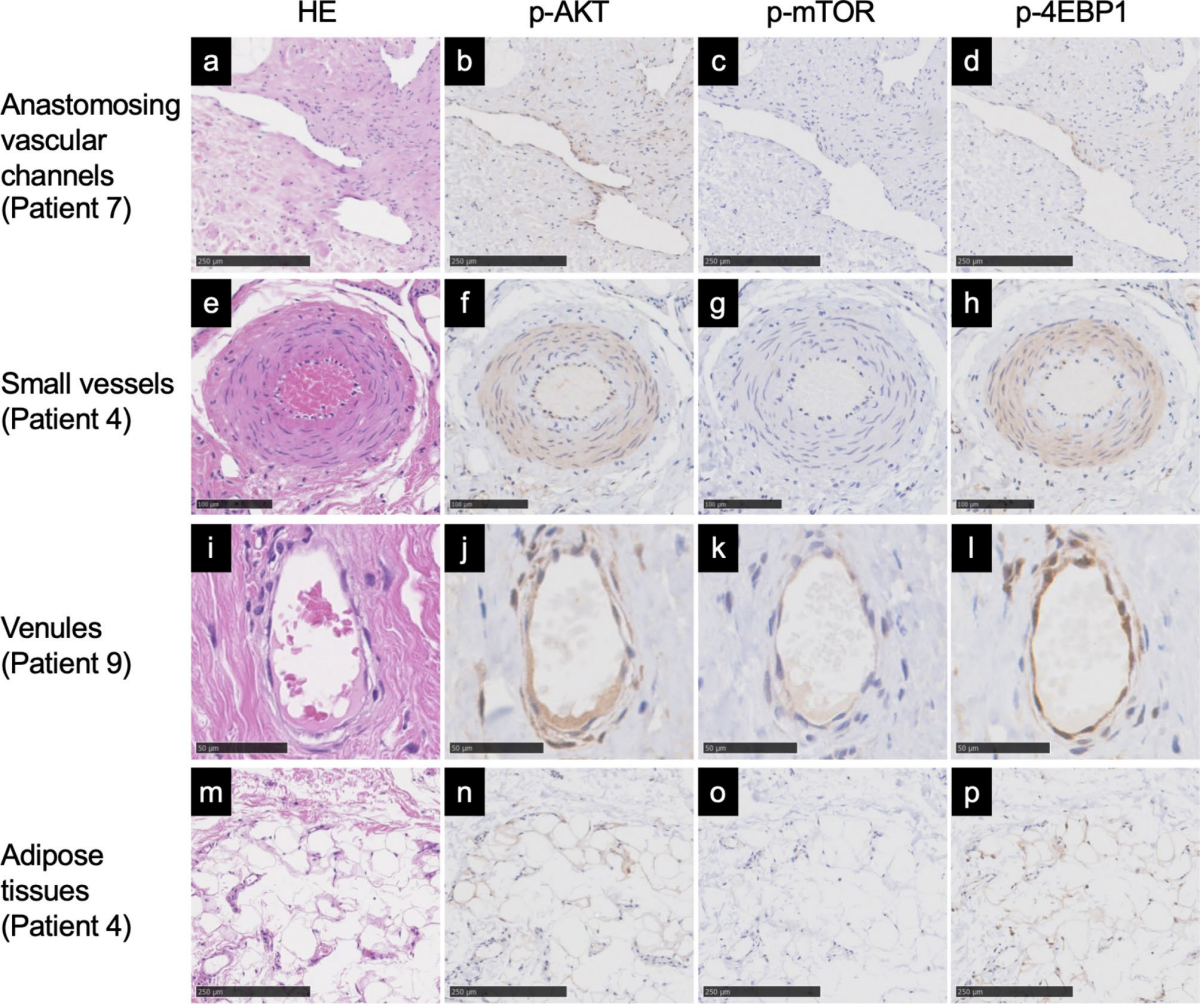
Histology and immunohistochemical analysis of the PI3K/AKT/mTOR signaling pathway in the serial sections of patients with Klippel–Trenaunay syndrome. Representative images of anastomosing vascular channels (**a**-**d**), small vessels (0.1–1.0 mm diameter) (**e**-**h**), venules (10–100 μm diameter) (**i**-**l**), and adipose tissues (**m**-**p**). Staining for hematoxylin and eosin (HE) (**a**, **e**, **i**, **m**), p-AKT (**b**, **f**, **j**, **n**), p-mTOR (**c**, **g**, **k**, **o**), and p-4EBP1 (**d**, **h**, **l**, **p**). Cytoplasmic intensity of the immunohistochemical stains graded as positive (**b**, **d**, **f**, **h**, **j**, **k**, **l**, **n**, **p**) and negative (**c**, **g**, **o**). Scale bars: **a**-**d**, **m**-**p** = 250 μm, **e**-**h** = 100 μm, **i**-**l** = 50 μm

The expression of D2-40 in lymphatic endothelial cells was confirmed in 6 patients. Anastomosing vascular channels were found in all 14 patients with positive stains for p-AKT (*n* = 12), p-mTOR (*n* = 7), and p-4EBP1 (*n* = 14); small vessels were found in 11 patients with positive stains for p-AKT (*n* = 10), p-mTOR (*n* = 1), and p-4EBP1 (*n* = 11); venules were found in all 14 patients with positive stains for p-AKT (*n* = 14), p-mTOR (*n* = 10), and p-4EBP1 (*n* = 14); and adipose tissues were found in all 14 patients with positive stains for p-AKT (*n* = 8), p-mTOR (*n* = 1), and p-4EBP1 (*n* = 12). Positive stains for p-AKT, p-mTOR, and p-4EBP1 were also found in two patients with undetected *PIK3CA* variants in vessels and adipose tissues. Representative images of hematoxylin and eosin, p-AKT, p-mTOR, and p-4EBP1 stains are shown in Fig. 3. The immunohistochemical analysis results for all patients are shown in Table 3.

**Table 3:** Immunohistochemical analysis of the lymphatic endothelial marker D2-40 and the PI3K/AKT/mTOR signaling pathway

		Anastomosing vascular channels				Small vessels				Venules				Adipose tissues		
Patient	D2-40	p-AKT	p-mTOR	p-4EBP1		p-AKT	p-mTOR	p-4EBP1		p-AKT	p-mTOR	p-4EBP1		p-AKT	p-mTOR	p-4EBP1
1	+	+	+	+		+	−	+		+	+	+		+	+	+
2	+	+	−	+		+	−	+		+	+	+		+	−	+
3	−	+	+	+		+	−	+		+	+	+		+	−	+
4	+	+	−	+		+	−	+		+	+	+		+	−	+
5	+	+	−	+		+	−	+		+	−	+		−	−	+
6	+	+	+	+		None	None	None		+	−	+		+	−	−
7	−	+	+	+		+	+	+		+	−	+		−	−	−
8	−	+	+	+		+	−	+		+	+	+		−	−	+
9	−	+	+	+		+	−	+		+	+	+		−	−	+
10	−	+	+	+		None	None	None		+	+	+		+	−	+
11	−	+	−	+		+	−	+		+	+	+		+	−	+
12	+	−	−	+		−	−	+		+	+	+		−	−	+
13	−	−	−	+		+	−	+		+	+	+		−	−	+
14	−	+	−	+		None	None	None		+	−	+		+	−	+

The expression of D2-40 in lymphatic endothelial cells was graded as positive (+) or negative (−). The cytoplasmic intensity of the immunohistochemical stains was graded as positive (+) or negative (−) in p-AKT, p-mTOR, and p-4EBP1. None, no small vessels observed

## Discussion

This study investigated the largest cohort of molecularly diagnosed patients with KTS in an Asian population. Using archival FFPE tissues, we identified *PIK3CA* variants in 85.7% of our cohort with KTS, 66.6% of which were the hotspot variants E542K, E545K, H1047R, and H1047L. The non-hotspot variants Q546K and Q546R were also identified, despite being rare in patients with vascular malformations. To our knowledge, Q546K was previously unreported in patients with KTS but was found in a patient with a fibro-adipose vascular anomaly [6] and in 3 patients with LM [8, 52]. Q546R has been reported in a patient with KTS [7] as well as a patient with LM [8] (Fig. 2). Our mutational findings were in line with those of patients with KTS in Western populations [3, 6, 8].

The PI3K catalytic subunit p110α encoded by *PIK3CA* has five domains: a C2 domain, a helical domain, a kinase domain, an N-terminal adapter-binding domain, and a Ras-binding domain (Fig. 2) [56]. In many cancers, mutations are found throughout the entire p110α protein, except for the Ras-binding domain, including the following hotspots: E542 and E545 in the helical domain and H1047 in the kinase domain [17]. The *PIK3CA* variants in PROS, including KTS, have a similar profile to that in cancers (Table 2), and an association of hotspot variants with more severe hypertrophy has been suggested, with milder hypertrophy linked to rarer non-hotspot variants [7, 27, 30]. However, in our cohort, patients who had non-hotspot variants did not exhibit milder hypertrophy compared with those who had hotspot variants. Thus, a meta-analysis should be conducted to clarify any potential genotype–phenotype correlations in this rare disease. To that end, molecular diagnoses may prove helpful in providing prognostic information on clinical manifestations [20].

Molecular genetic testing for the diagnosis of PROS requires clinically affected tissues, preferably fresh frozen tissues [4]. Testing can be performed using FFPE tissues; however, unlike fresh frozen tissues, DNA obtained from FFPE tissues can vary widely in terms of quality [19]. NGS with the use of a highly sensitive amplicon-based targeted sequencing method can identify low-level mosaicism from DNA extracted from widely available archival FFPE tissues [20]. Although digital droplet PCR is a simple and highly sensitive and specific method for the detection and quantification of targeted DNA variants, entire exons must be sequenced by NGS in order to capture all the coding single-nucleotide variants as well as small insertion and deletion variants in rare diseases [4]. Detection of rare variants remains a challenge because of the error-prone nucleotide changes resulting from sequencing errors. NGS combined with molecular barcodes can eliminate false-positive variants and enable detection thresholds of 0.1% VAF [57].

Using archival FFPE tissues, we were able to compare the genotype and histology of the lesions. Immunohistochemistry revealed that the vessels in all 14 patients and the adipose tissues in 13 patients expressed p-AKT, p-mTOR, and/or p-4EBP1 (Fig. 3; Table 3), indicating enhanced activation of the PI3K/AKT/mTOR pathway in mesenchymal tissues compared with that in normal tissues in these patients with KTS. These findings are in line with previous reports of *PIK3CA* variants detected in adipocytes in PROS [3] and abnormal vessels detected in fibro-adipose vascular anomaly [58]. However, 2 patients (patients 13 and 14) with undetected *PIK3CA* variants showed some expression of p-AKT, p-mTOR, and/or p-4EBP1 in vessels and adipose tissues. Neither had geographic CM, histopathological LM, or macrodactyly of the foot. Patient 13 can be diagnosed with CVM and congenital nonprogressive limb overgrowth [59] caused by somatic *GNA11* mutation [60]. Patient 14 might have common VM caused by a somatic *TEK* mutation [61] encoding TIE2 upstream of the PI3K/AKT/mTOR pathway.

To date, fewer than 30 different *PIK3CA* gene variants have been reported in PROS, five of which—C420R, E542K, E545K, H1047R, and H1047L—have been shown to be recurrent [5]. As for the *PIK3CA* variants in patients with KTS [3, 6–9], Kurek et al. reported H1047R in 3 of 15 patients [3], while Luks et al. reported E542K in 3, E545K in 9, E545G in 1, H1047R in 6, and H1047L in 1 of 21 patients [6]. Kuentz et al. reported G364R in 1, E542K in 1, E545K in 2, Q546R in 1, and H1047L in 1 of 13 patients [7]. Brouillard et al. reported E110del in 1, and E545K in 3 of 4 patients [8]. Nozawa et al. reported E542K in 2, E545K in 5, and H1047R in 1 of 10 patients [9].

The therascreen^®^ PIK3CA RGQ PCR Kit was developed to be a companion diagnostic tool for 11 *PIK3CA* gene variants: C420R, E542K, E545A, E545D, E545G, E545K, Q546E, Q546R, H1047L, H1047R, and H1047Y. It uses genomic DNA extracted from FFPE or circulating tumor DNA isolated from plasma in patients with breast cancer. Patients with advanced or metastatic breast cancer who test positive for the presence of one or more *PIK3CA* variants are eligible for treatment with the PI3K inhibitor alpelisib [62]. With a limit of detection from 2.4 to 14.04% VAF [63], the therascreen kit would not have detected five cases in our cohort because of the low VAF (C420R with 1.6% VAF; E542K with 3.3% VAF; Q546R with 3.9% VAF and 5.9% VAF, respectively; and H1047R with 1.6% VAF) as well as one case with the Q546K variant, which is not targeted by the kit.

Our findings have potential implications for the treatment of patients with KTS using inhibitors of the PI3K/AKT/mTOR signaling pathway, which have shown promising results with mTOR inhibitor sirolimus [64, 65], pan-AKT inhibitor miransertib [66], and selective class I PI3K inhibitor alpelisib [67] or taselisib [68]. It is thus critical to obtain more detailed information regarding specific variants in order to identify of the options for targeted treatment [69].

This study has some limitations. First, we evaluated only patients with KTS who were diagnosed based on the triad of CM, VM, and hypertrophy of the affected limb [2], so there was no controlling for vascular malformations except KTS in the immunohistochemical analysis. Second, we performed targeted sequencing of *PIK3CA* gene coding sequences using a panel consisting of amplicons with an overall coverage of 87.9%, so genes not on the panel would have been missed.

## Conclusions

We identified *PIK3CA* variants in 12 of 14 patients (85.7%) with KTS by using archival FFPE tissues, and 8 of these patients had the following hotspot variants: E542K, E545K, H1047R, and H1047L. The rarer non-hotspot *PIK3CA* variants Q546R and Q546K were also identified in 3 patients. Amplicon-based targeted NGS was able to identify low-level mosaicism from low-input DNA extracted from FFPE tissues, suggesting its potential as a diagnostic option for personalized medicine.

## Methods

### Patients

This retrospective study involved Japanese patients with vascular malformations with lower limb hypertrophy who underwent resection of the vascular malformations at Tonan Hospital between 2011 and 2020. Of the 17 patients identified, 14 provided written informed consent and were included in the analysis. KTS was diagnosed based upon the triad of CM, VM, and hypertrophy of the affected limb [2]. In younger patients, VM was often less conspicuous but was diagnosed based on veins that were subtly dilated relative to the unaffected limb [70].

The patients’ medical charts were reviewed, and the following demographic information and medical history data were collected: sex, date and age at resection, lesion resected, type of tissues in resected specimens, clinical photographs, and radiologic studies. Geographic CMs were defined as those with sharply demarcated borders and saturated dark red/purple color throughout the entire stain [70].

Prior to resection, all patients underwent MRI and color duplex ultrasound to evaluate the characteristics, distribution, and extent of the lesions. Vascular malformations were diagnosed based on the clinical history as well as the physical examination, ultrasonography, and MRI findings. LLD was evaluated in terms of length and girth according to teleoroentgenography and cross-section on MRI, respectively. LLD in terms of length was defined as 5 mm longer compared with the unaffected limb in children and 1 cm longer in adults, while LLD in terms of girth was defined as 10% greater in cross-sectional area compared with the unaffected limb [71].

### DNA extraction

Surgical specimens were fixed with 10% buffered formalin and embedded in paraffin. Affected tissue was retrieved from archived FFPE tissue blocks. QIAamp DNA FFPE Tissue Kit (Qiagen, Germantown, MD) was used to extract genomic DNA from FFPE tissues. The positive and negative control DNA from the FFPE *PIK3CA* Reference Standard (E545K, HD112; H1047R, HD599; wild-type [WT], HD320; Horizon Discovery, Cambridge, UK) were extracted using a Maxwell RSC FFPE DNA Kit (Promega, Madison, WI). The concentration and quality of the extracted DNA were assessed using a 2200 TapeStation system with the Genomic DNA ScreenTape (Agilent, Santa Clara, CA) and a Qubit 3.0 Fluorometer with a Qubit dsDNA BR Assay Kit (Thermo Fisher Scientific, Waltham, MA), respectively. DNA controls were prepared for each condition: 5%, 1%, 0.5% and 0.1% of E545K/ H1047R mixture diluted in WT for the positive controls and only WT for the negative control.

### Next-generation sequencing

We used an Ion AmpliSeq™ HD Made-to-Order Panel (IAH215884_374) to perform targeted sequencing of all *PIK3CA* gene coding sequences (3,607 bp). The panel consisted of 64 amplicons with an overall coverage of 87.9%. Deaminated cytosine residues were removed from 20 ng of the sample DNA and the target region was amplified using multiplex PCR with the Ion AmpliSeq™ HD Library Kit (Thermo Fisher Scientific). The primer sequence in the amplicon was partially digested, and the library was amplified using primers, to which barcode sequences were added using Ion AmpliSeq™ HD Dual Barcode Kit (Thermo Fisher Scientific). After purification of the library DNA, the concentration and size of DNA were checked, and the library was mixed. Emulsion PCR was performed to clonally amplify the library DNA on beads and then template beads were collected and sequencing reactions were performed on the Ion S5™ XL system (Thermo Fisher Scientific) using an Ion Chef 550 Chip Kit (Thermo Fisher Scientific).

### Bioinformatics analysis for detection of ***PIK3CA*** variant

The quality of the read data was checked, and the adapter sequences and poor-quality reads were removed. Then, the reads were mapped to reference sequences (hg19) using the torrent mapping alignment program of Torrent Suite ver. 5.16.1(Thermo Fisher Scientific) and variants were detected using Ion Reporter ver. 5.18 (Thermo Fisher Scientific). Annotation information was assigned to the detected variants. The thresholds for the main parameters of Ion Reporter’s variant detection were set as follows: Downsample to Coverage: 20,000; Minimum Allele Frequency of SNP (single nucleotide polymorphism), MNP (multiple nucleotide polymorphism), and INDEL (insertion or deletion of nucleotides): 0.05%; Minimum Variant Score of SNP and MNP: 6; Minimum Variant Score of INDEL: 10.

### Histology and immunohistochemistry

The serial FFPE Sect. (5 μm thick) were stained using immunohistochemical as well as hematoxylin and eosin stains for a lymphatic endothelial marker D2-40 (#916,606, 1:1,000, BioLegend, San Diego, CA), p-AKT (#4060, 1:75; Cell Signaling Technology, Danvers, MA), p-mTOR (#2976, 1:100; Cell Signaling Technology), and p-4EBP1 (#2855, 1:200; Cell Signaling Technology). The expression of D2-40 in endothelial cells was used to diagnose LM. Vessels were categorized into three groups according to type and/or size: anastomosing vascular channels [72], small vessels (0.1–1.0 mm diameter), and venules (10–100 μm diameter). Vessels and adipose tissues were evaluated in terms of the cytoplasmic intensity of immunohistochemical stains graded as positive or negative by two independent observers. The connective tissues surrounding the lesions were used as a control.

### Electronic supplementary material

Below is the link to the electronic supplementary material.

Supplementary Material 1

## Data Availability

The datasets generated and analyzed during this study are available from the corresponding author upon reasonable request.

## Abbreviations

CM: capillary malformation
CVM: capillaro-venous malformation
CLVM: capillaro-lymphatico-venous malformation
4EBP1: eukaryotic translation initiation factor 4E-binding protein 1
FFPE: formalin-fixed paraffin-embedded
KTS: Klippel–Trenaunay syndrome
LLD: lower limb discrepancy
LM: lymphatic malformation
MRI: magnetic resonance imaging
mTOR: mammalian target of rapamycin
NGS: next-generation sequencing
PI3K: phosphatidylinositol 3-kinase
PROS: *PIK3CA*-related overgrowth spectrum
VAF: variant allele frequency
VM: venous malformation
WT: wild-type
